# Case report—Every thoracic surgeon's nightmare: cardiac and lung perforation during placement of Nuss bar for pectus excavatum

**DOI:** 10.3389/fped.2023.1241273

**Published:** 2023-09-01

**Authors:** Federico Beati, Simone Frediani, Valerio Pardi, Ivan Aloi, Arianna Bertocchini, Antonella Accinni, Alessandro Inserra

**Affiliations:** General and Thoracic Pediatric Surgery Unit, Bambino Gesù Children’s Hospital, IRCCS, Rome, Italy

**Keywords:** pectus excavatum, Nuss, lung perforation, cardiac perforation, children

## Abstract

**Introduction:**

The prevalence of life-threatening complications (LTCs) related to the minimally invasive repair of pectus excavatum (MIRPE) is unknown and underreported. The aim of this study is to contribute to the real prevalence of these rare but dramatic complications and show what went wrong in order to prevent it in the future.

**Case presentation:**

A 15-year-old boy affected by pectus excavatum with severe asymmetric deformity of the chest wall was evaluated for elective corrective surgery. Preoperative computed tomography showed a Haller index of 5.7 and a correction index of 0.40. MIRPE was performed under right video-assisted thoracoscopy. Cardiac arrhythmias occurred after placement of the bar introducer. The introducer was removed, and massive bleeding was noted. Emergency Clamshell thoracotomy was performed, and cardiac surgeon was alerted immediately. A first pulmonary wound was found and controlled. Two cardiac lacerations were found: on the interventricular wall and on the right atrium. Under cardiopulmonary bypass, cardiac lacerations were sutured and other three pulmonary wounds were repaired. An urgent fasciotomy was performed for compartmental syndrome of the right lower art after femoral cannulation. Pulmonary distress occurred; the patient was admitted on ECMO (ExtraCorporeal Membrane Oxygenation) in intensive care unit. Right lower lobectomy was carried out on the fifth postoperative day due to massive pulmonary bleeding requiring temporary tracheostomy. The patient was discharged to rehabilitation after 3 months with no brain injuries, minor hearing loss, and tracheostomy.

**Conclusion:**

We want to maintain the high alertness required for this procedure. Reporting these scaring complications contributes to the real prevalence of LTCs. We suggest the use of bilateral thoracoscopy and crane elevator in severe sternal defects. We also suggest to have a cardiac surgeon available in the hospital owing to cardiac perforation.

## Introduction

Minimally invasive repair of pectus excavatum (MIRPE) was described by Nuss for the first time in 1998 (1), and it is considered the gold standard for surgical repair of pectus excavatum (PE). It is universally accepted as a safe and effective procedure (2), even if an increasing number of life-threatening complications (LTCs) are reported during both bar placement and removal with dramatic consequences on patients and their families (3–6).

The aim of this study was to share our dramatic complication during MIRPE, cardiac perforation (CP) associated with pulmonary injury, to contribute to the literature the real incidence of this major complication and to prevent it in the future.

## Case report

**Figure 1:**
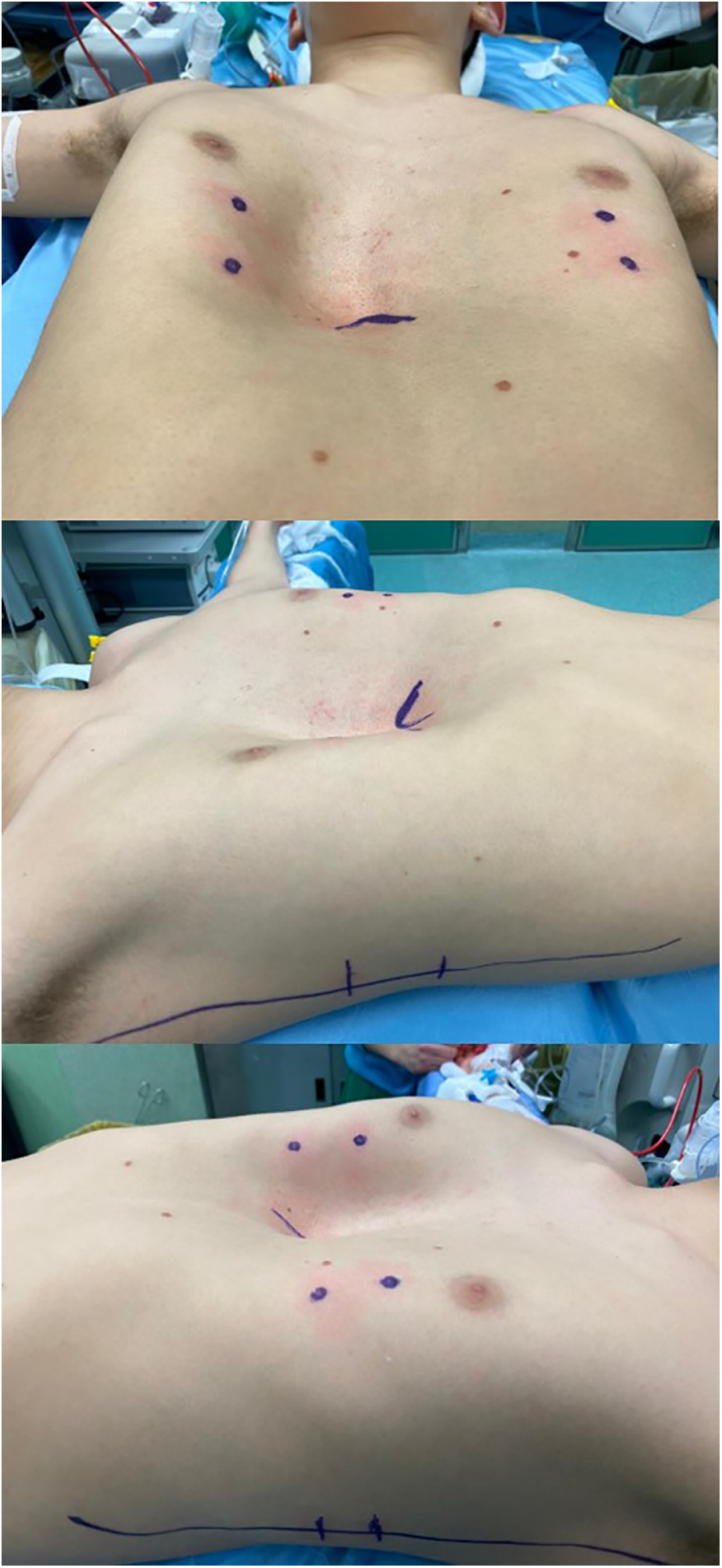
Preoperative valutation.

A 15-year-old boy affected by PE with severe asymmetric deformity of the chest wall was evaluated for elective corrective surgery (Figure 1). He was asymptomatic except for shortness of breath during sport activity. Preoperative electrocardiogram, echocardiography, and pulmonary function tests at rest were normal.

Preoperative CT showed a severe thoracic defect with a Haller index of 5.7 and a correction index of 0.40. He also had a left-sided rotation of the sternum.

After an exhaustive discussion with the patient and his parents, comprehensive of all potential complications, including cardiac injury, a preoperative informed consent for MIRPE procedure was obtained from the parents.

Under general anesthesia, a right thoracoscopy was performed showing a severe sternal defect. We decided to use a vacuum bell to create a space between the sternum and the heart, and a 5-mm port was placed into the right hemithorax. Blunt dissection was done from the right to the left pleural cavity, under vision, using a Johann grasper. The introducer was passed through the retrosternal tunnel under thoracoscopic vision emerging into the left pleural cavity, and arrhythmia and hypotension were noted by the anesthesiologist. The introducer was immediately removed, and massive bleeding was seen coming from the tunnel. A major cardiac or pulmonary injury was suspected. The cardiac surgeon was immediately notified, and a clamshell thoracotomy was performed. Pulmonary bleeding was identified on the lower right lobe, initially controlled with a vascular clamp, and a concomitant pericardium tamponade was noticed. Open cardiac massage was performed while cardiopulmonary bypass was started by inserting cannulas into his right femoral vessels. Two cardiac lesions were seen: one on the anterior part of the interventricular wall, very close to the right coronary vessel, and one on the right atrium. An additional cannula was inserted into the lower vena cava, and right atriotomy was performed; the two wounds were repaired with non-absorbable sutures. On the right lower pulmonary lobe, four lesions were seen and repaired with non-absorbable sutures.

**Figure 2:**
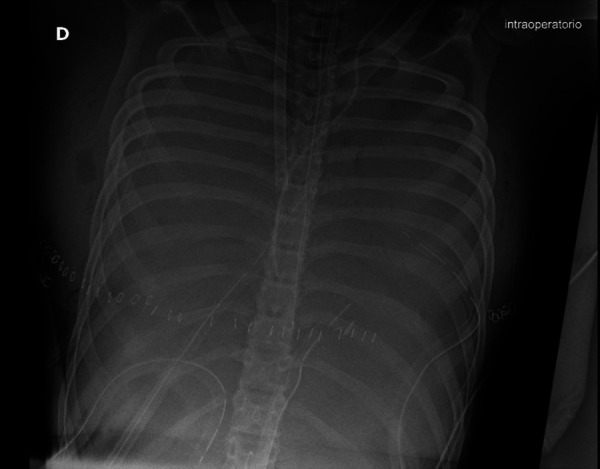
Intraoperative x-ray chest showed an ARDS syndrome.

The patient developed an acute hypoxemic respiratory failure, and intraoperative x-ray chest showed an ARDS (Acute Respiratory Distress Syndrome) syndrome (Figure 2). The cardiac surgeon decided to shift the cardiopulmonary bypass into veno-arterial ECMO assistance. At the end of cardiac surgery, a weak pulse and skin discoloration were observed on the right limb, and an emergency fasciotomy was performed in the lateral and posteromedial compartments. The patient was admitted to the intensive care unit on ECMO.

**Figure 3:**
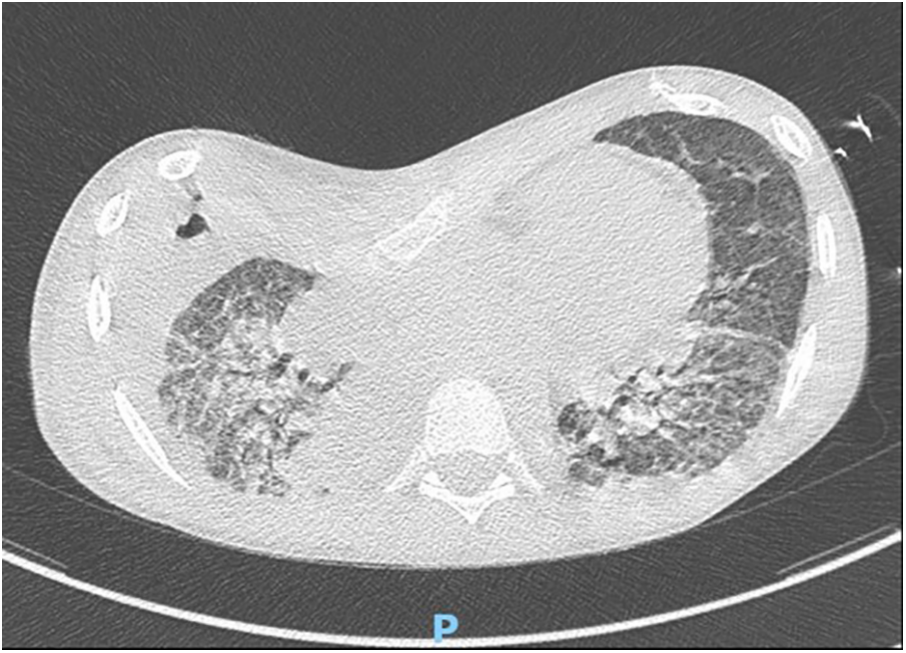
Persistence of ARDS and pulmonary bleeding in fifth postoperative days.

A right lower lobectomy on ECMO assistance was necessary due to the persistence of ARDS and pulmonary bleeding on the fifth postoperative day (Figure 3). A temporary tracheostomy was performed on the 15th postoperative day because of prolonged intubation. Delayed primary wound closure was possible after negative pressure wound therapy for the right lower limb.

The patient was discharged to rehabilitation unit after 3 months with no brain injuries, minor neurosensitive hearing loss, and a temporary tracheostomy tube. After 6 months of rehabilitation, the tracheostomy tube was removed without any complications.

## Discussion

We report the most scaring LTC during MIRPE procedure: CP and lung injury.

A recent survey of the Chest Wall International Group (CWIG) from 1998 to 2016 reported 27 published cases and 32 unreported LTCs including cardiac perforation, hemothorax, major vessel injury, and lung injury. They identified seven unpublished death cases (five secondary to cardiac injury and two related to gastro-intestinal adverse events). There were seven cases of major complications with bar removal (reported and non-reported) with two lethal outcomes (7).

Analyzing the literature, the first case of cardiac perforation was described by Moss et al. in 2001 during Nuss procedure in an 8-year-old boy. He required urgent sternotomy with right atrial and right ventricle repair followed by tricuspid valve repair on cardiopulmonary bypass (8).

Gips et al. (9) reported the first case of death during PE correction with a highly modified Ravitch repair procedure. Autopsy findings revealed three wounds in the right atrium and ventricle, including one in the right coronary artery.

In 2011, Becmeur et al. proposed a literary review of major complications during the Nuss procedure. He described 16 cases of LTC, 11 of which were cardiac perforations. Among these, two patients died and one survived with severe hypoxic ischemic encephalopathy (10). Data related to LTC for PE correction between 1998 and 2016 were obtained in a recent survey by Hebra et al. (7) reporting a total of 27 cardiac injuries, 15 of those were unreported previously. Among other LTCs, they reported aortic or major vascular injuries, massive hemo/pneumothorax, cardiac or major vessel occlusion, and sternal erosion.

The last case of cardiac perforation was reported by Senica et al. (11) in 2022 in which the metal bar entered from the right atrium and passed through the interventricular wall and left ventricle.

Several tips and tricks have been described over the years to prevent LTC using bilateral thoracoscopy in selected cases and sternal elevator techniques such as vacuum bell (12), Kent retractor (13), Rultract® retractor (14), and subxiphoid incision (15). The critical step is to create a “safe” space between the pericardium, a posterior part of the sternum, and make the blind dissection easier. The retrosternal space is safe when the tip of the introducer is always under direct view. We usually utilize the vacuum bell to elevate the sternum, as it was our preference even in severe defects. This technique provided a partial correction of the defect, sufficient to pass the bar introducer but probably not creating a safe retrosternal space. Bilateral thoracoscopy is widely described, but we did not introduce a contralateral trocar because we believed that a severe sternal rotation could not allow a correct view of the left pleural cavity. This probably contributed to the cardiac lesion.

Pulmonary bleeding is a described complication during bar removal (16) but is not frequent in bar placement. We think that pulmonary lacerations could be caused by immediate removal of the bar introducer without any thoracoscopic vision. It also could be iatrogenic damage during the emergency clamshell procedure.

Analyzing the literature, sternotomy is preferred in this type of emergency to better control bleeding. In our case, the thoracic surgeon was more confident with clamshell thoracotomy, which also allowed a better control of pulmonary bleeding.

The patient survived because of the immediate assistance of an expert thoracic surgeon and the cardiothoracic team.

## Conclusions

The task of conveying these intricate complexities within the international literature can prove challenging; yet, we assert that it is imperative to apprise all surgeons, regardless of their expertise, about potential errors that may have life-threatening consequences. Due to the presence of a significant defect, it would be advisable to employ a sternal crane and bilateral thoracoscopy techniques in order to get improved visual outcomes, despite their deviation from our customary approach. We also suggest having a cardiac surgeon on call warned when a Nuss procedure is performed, and that this type of surgery should be performed by a surgeon or a pediatric surgeon with experience in emergency thoracic procedures.

## Data Availability

The original contributions presented in the study are included in the article/Supplementary Material, further inquiries can be directed to the corresponding author.
